# Potential alteration of COVID-19 by beta-mercaptoethanol

**DOI:** 10.2217/fmb-2020-0142

**Published:** 2020-09-21

**Authors:** Robert E Click

**Affiliations:** ^1^Altick Associates, 2000 Maxwell Drive, Hudson, WI 54016, USA

**Keywords:** covid-19, herd immunity, beta-mercaptoethanol (BME), T- and B-cell enhanced functions, suppressor cell inactivation

When and how the COVID-19 pandemic caused by SARS-CoV-2 ends is the topic of substantial debate. Potential options to return societies back to a somewhat normal lifestyle appear to depend upon achieving one of the following: develop a vaccine; discover a drug that will disrupt the viruses’ survival/reproductive processes; and a combination of the other two. Development of any of these may require a considerable amount of time without any assurances. Hardly an inevitable situation.

While following the chatter on TV and internet, an interview with Harvard’s Professor Lipsitch, on the concept of ‘herd immunity’ was very intriguing. Almost immediately I pondered: Why is this concept not more seriously considered as a means to combat COVID-19? Based on many group’s attitude to disregard preventative suggestions of infectious disease experts, along with the relaxation of government regulations, mini-herd immunity has essentially already begun. From an outsider’s perspective, such an approach: would take considerably less time to implement than that projected to develop a vaccine; may not require isolation of virus or viral-constituents; and if the severity between initial infection and pathological consequences were minimized/curtailed, the death rate would likely be considerably less. It could be argued that now is a perfect opportunity to explore the means of curtailing the severity of accompanying pathological events.

A search of the literature for a magic, antiseverity protocol resulted in multiple articles that described means to alter various aspects of viral diseases. One especially interesting group is composed of antioxidant, low-molecular-weight (LMW) thiols; the most thoroughly studied for human use is *N*-acetylcysteine (NAC) and to a much lesser extent, beta-mercaptoethanol (BME). In one study, the proliferation of *in vitro*, nonstimulated, T-cell colonies present in PBMC of ‘normal’ (186%) and AIDS (234%) individuals was equally enhanced in the presence of NAC; results that contrasted to the greater increase in colonies from AIDS (401%) than normal (168%) individuals when BME replaced NAC in the culture medium. The difference in growth of AIDS cells was attributed to BME more effectively inactivating suppressor cells because plasma of AIDS patients equally inhibited growth of control T cells in the presence (48%) or absence (52%) of BME [1]. In a separate study [2], pretreatment of anergic CD4^+^ cells of HIV-infected patients with a NAC/BME admixture enhanced the CD3-mAb stimulated responses of ‘high’, but not ‘low’, responders. Additional investigations by others indicated that: NAC therapy of HIV-infected individuals improved their survival, which was associated with reversal of T-cell glutathione deficiencies [3]; modulation of cell surface redox events suppressed the cellular entry of HIV [4]; intracellular HIV replication was suppressed by NAC [5,6]; and inhibitory factors produced by AIDS T cells that specifically blocked B-cell antibody production *in vitro* were inactivated by BME [7].

It was somewhat surprising that a survey in April 2020 on behalf of the Oxford COVID-19 Evidence Service Team, failed to find any evidence that indicated NAC possessed COVID-19 specific benefits [8]. However, it is easy to argue that it is premature for data from any such study (if it exists) to be published. Indeed, since submission of the present manuscript, a perspective was published on why NAC should be considered as a therapeutic option for COVID-19 [9]. Other recent information indicates that among 827 COVID-19 patients, 76% had low total, 76% low CD4^+^ and 72% low CD8^+^ T-cell counts [10]. Further, the CD4^+^ and CD8^+^ T-cell counts were lower in ICU than non-ICU patients suggesting that their T cells were exhausted. The authors concluded [10]: “*Taken together, new therapeutic measures are needed for treatment of COVID-19 ICU patients.*” Considering that both **intracellular** and **cell surface** thiols of adaptive and innate immune cells are modifiable by external LMW thiols [11,12], new therapeutic areas of interest may include: alteration of undetectable antigenic epitopes; increase insufficient effector cells (precursor or nonexhausted) to overcome suppressor cell functions; strengthen TCR-peptide/MHC interactions; modify poor costimulatory signals; Ir-genes; and others [13–16]. The present report will summarize data that indicates BME is a better thiol choice than NAC, because it is more potent, has broader pleotropic effects, requires up to a 100-fold lower dose, is recycled without being modified or biologically incorporated, and lacks some of the adverse insults of NAC [17,18]. These attributes suggest that it is time to assess whether BME has any benefits for human diseases irrespective of its designation as a ‘poison’. The benefits of daily consumption of mg of BME for entire lives of many different strains of mice by three different research groups [19–24] suggest that functional levels of BME are not poisonous and (as yet) have not been shown to cause any serious side effects. The present COVID-19 pandemic offers an ideal opportunity to test BME for safety/benefits in humans as dosage, formulation and packaging are established. Furthermore, considering the reality of a COVID-19 vaccine, one need only hope that the experience with AIDS ‘vaccines’ will not be encountered with COVID-19. Such a possibility should encourage multiple, including unconventional, investigations.

## BME alterations of immune functions

Until recently, modes which alter immune processes have not been very successful as therapeutic cancer treatments. With the discovery of immune checkpoint inhibitors, this lull seems to be evaporating. To gain an appreciation of BME benefits, a short history should be enlightening as it relates to the present-day pandemic.

### In vitro

Numerous investigations on adaptive immunity followed the initial reports in the ‘70s that both humoral [25–27] and cell mediated [28–31] responses *in vitro* were enhanced multifold by LMW thiols, with BME and dithiothreitol (DTT) being the most potent. Similar antibody enhancements were described soon thereafter for cysteamine [32] and α-thioglycerol (α-TG) [33]. In some cases, BME enhanced primary antibody synthesis >100-fold [25,26] and proliferation/differentiation of primary and secondary alloantigen specific cytotoxic T lymphocytes >50-fold [34,35]. Over the ensuing years, alterations of *in vitro* processes by BME were confirmed and extended by hundreds of investigators. The magnitude of enhancements by, plus dosages of, these thiols differed enormously regardless of whether they were added to serum-free culture medium or medium supplemented with homologous or heterologous sera. The most potent, BME, cysteamine, DTT and a-TG, are unique in that they are **not** endogenous bioconstituents. Further, the reduced and oxidized forms of BME, cysteamine and DTT increased the number of sRBC antibody producing B cells equally [32]; the mechanism of DTT, however, differed from that of BME and cysteamine in that enhancement was via T-cell activation of B cells rather than direct activation of B cells. This mechanism was similar to that of lipoic acid [36] and NAC [37] in that none facilitated uptake of extracellular cystine.

In addition to the importance of adaptive immunity, innate type I and II NK cells are also major participants in viral infections and cancer [38]. NK cells can be activated by a number of receptors, including NKG2D, a disulfide-linked homodimer that recognizes a number of ligands [39]. It is not surprising, therefore that cell surface thiols covalently coupled with the nonpenetrating reagent, monobromotrimethyl-ammoniobimane, prevented a line of cloned, murine, NK cells to bind to and lyse target cells [11]. Subsequently, this was extended to include a thiol role for lytic processes of human NK cells [40]. Further, an *in vitro* requirement for BME was found for induction of lymphokine-activated killer cell activity of rat cultures [41] and for enriched human NK cells [42]. In the latter, both thiol deprivation and H_2_O_2_ treatment resulted in oxidative stress that increased intracellular ROS levels. This altered internalization of IL-2 and maturation of the lytic mechanism by inhibition of *FasL* induction. Based on these *in vitro* results the authors hypothesized that thiol-reducing compounds may be a physiological **requirement** for maintenance of optimal NK functions under *in vivo* oxidative conditions [42–44]. A further effect of BME was its requirement for NK cell generation in nonconditioned, long-term bone marrow cultures directly from enriched NK precursors present in rat marrow [45].

### In vivo

As might be expected from the extensive literature on LMW thiol alterations of immune responses *in vitro*, investigations on *in vivo* disease processes soon followed. One of the first areas to be investigated was age-associated, declines of both primary and secondary antibody synthesis, which were slowed/reversed by *in vitro* [46–51] or *in vivo* [19,20] ‘BME-therapy’. The declines of these adaptive immune responses were caused by increases in nonadherent suppressive T cells [46,47]. BME [52] and glutathione [53] also reversed *in vitro*, the age associated, declines of immune functions of rats. Makinodan, collaborators and others reported that innate immune functions of mice also declined with age; declines postulated to be a consequence of multiple factors that included: the absolute loss of NK cells due to reduced generation in the bone marrow; a decreased capacity of NK cells to bind to target cells; and increased adherent cell suppressor functions [54–56] – the latter cells differed from those that suppressed antibody synthesis [46,47]. Further, NK functions as well as immune responses of old mice were enhanced by other thiols added to their feed [57,58], including NAC and thioproline (BME was not tested). Stimulation by more classic antioxidants, alpha-tocopherol (vitamin E) and ascorbic acid, was less than those by the two sulfur containing antioxidants [57].

### Role of cysteine

Finally, many functions altered by exogenous LMW thiols (but not lipoic acid or NAC) are directly dependent upon the availability of cysteine/cystine. This dependence was first noted when BME enhancement of primary antibody formation to sRBC did not occur when cystine was omitted from the culture medium [59]. There was a specific LMW thiol rank-order in uptake of (^35^S)-cystine by splenocytes – the largest was by BME, followed by DTT and lastly by cysteamine [60]. Not surprising, the increase in the number of specific IgM producing B cells followed the same rank order. A steady state of replenishment of intracellular cysteine by BME was reached within 24 h by the two–fivefold enhanced rate of uptake [59]; a consequence of nonenzymatic formation of a mixed disulfide [60] taken up mainly via the L system, a transporter system for neutral alpha-amino acids such as leucine. The intracellular, mixed disulfide underwent rapid reduction to cysteine and BME; the latter escaped into the extracellular fluids where it was available to continue the cycle.

Depletion of intracellular cysteine was not unexpected since lymphocytes lack cystathionase, which converts methionine to cysteine and they lack an intact xc-transporter system to directly acquire cystine, the prominent bio-form, from liquid surroundings. Under such constraints, cysteine replacement is dependent upon the transport by the alanine, serine, cysteine (ASC) system of minuscule amounts not yet oxidized to the more stable disulfide form, cystine. These inadequacies are in part compensated for by uptake of cystine by APC/dendritic cells (both possess an intact xc-transporter system) which is reduced and exported as cysteine. Unfortunately, myeloid-derived suppressor cells further exacerbate availability by sequestering cystine via their xc-transporter system, **but** because they lack the ASC transporter, do not export cysteine [61,62]. Consequently, the lowered amount of cystine available for conversion to cysteine by APC/dendritic cells results in less extracellular cysteine in the surrounding milieu [37,61–66]. Failure to adequately replenish intracellular cysteine, the rate limiting component for GSH synthesis during lymphocytic stimulation, also resulted in depletion of glutathione (GSH). The consequence was lymphocytes became unable to proliferate (exhaustion?) to further stimulation. More recent findings added a significant modification, namely neither depleted glutathione nor cysteine alone is the cause of reduced T-cell proliferation, but they become rate limiting only in the absence of endogenous LMW thiols [67].

## Summary

Results presented herein demonstrating BME alters immune functions at very low concentrations, suggest it may safely curtail the severity of COVID-19 disease and prevent deaths by enhancement of antiviral, immune responses while also blocking suppressor cell activities; a hypothesis that should be easy to assess in a highly populated herd immune environment, such as a meat processing company or a nursing home, that would satisfy appropriate statistical validation. Input is encouraged from anyone with clinical trial expertise wishing to assess benefits of BME on COVID-19 disease.
